# Recognizing Flagellate Erythema in Skin of Color: A Case of Shiitake Dermatitis

**DOI:** 10.7759/cureus.55437

**Published:** 2024-03-03

**Authors:** Joseph Gofman, Lucas Shapiro, Merrick D Elias

**Affiliations:** 1 Dermatology, Nova Southeastern University Dr. Kiran C. Patel College of Osteopathic Medicine, Fort Lauderdale, USA; 2 Dermatology, Nova Southeastern University Dr. Kiran C. Patel College of Osteopathic Medicine, Clearwater, USA; 3 Dermatology, Elias Dermatology, Pembroke Pines, USA

**Keywords:** skin of color, shiitake, hyperpigmentation, dermatitis, erythema, flagellate

## Abstract

Flagellate erythema, also known as flagellate dermatitis, flagellate hyperpigmentation, or shiitake dermatitis, is a rare multifocal cutaneous eruption characterized by linear erythematous lesions similar to flagellation wounds. This case report details the progressive onset of flagellate erythema in a 31-year-old African American male presenting with pruritic, erythematous, hyperpigmented, linear lesions of the face, trunk, and upper extremities following his consumption of shiitake mushrooms. Classically, this eruption arises subsequent to the ingestion of raw or undercooked shiitake mushrooms. This case underscores the importance of clinical diagnosis, as the role of biopsy as a diagnostic tool is limited due to the nonspecific nature of histological findings. Therefore, proper diagnosis is reliant upon careful history taking, including dietary changes, initiation of any new medications, and progression of symptoms. Most cases are self-limiting, with eruptions persisting for up to three weeks. Treatment aims to provide symptomatic relief through topical corticosteroids and oral antihistamines, reducing associated pruritus and skin changes.

## Introduction

First described by Nakamura in 1977, flagellate erythema is a rare and distinctive cutaneous eruption [1]. This condition is characterized by grouped linear erythematous lesions, evocative of flagellation wounds - a reference to the act of whipping or lashing that yields linear cutaneous patterns of injury. Classically, this eruption arises subsequent to the ingestion of raw or undercooked shiitake mushrooms. Nevertheless, instances of flagellate erythema have also been documented in patients who consumed fully cooked shiitake mushrooms [2]. 

The cutaneous eruption of flagellate erythema is described as erythematous, hyperpigmented, linear papules, which present two hours to five days following ingestion of the mushrooms [1,3]. The initial rash is commonly preceded by pruritus and may include petechiae, plaques, vesicles, morbilliform, or pustular lesions lasting up to three weeks [3]. Systemic symptoms are uncommon, but they may include fever and diarrhea [4]. Diagnosis of suspected flagellate erythema is made clinically. Given the uniqueness of these flagellate eruptions, taking a detailed patient history is critical. Differential diagnoses include cutaneous eruptions secondary to chemotherapy with bleomycin, dermatomyositis, adult-onset Still's disease, and contact dermatitis. While a histological examination may aid in diagnosis, its utility is limited. Symptomatic treatment includes oral antihistamines and topical steroids for symptom relief, but most cases are self-limiting [1].

## Case presentation

A 31-year-old African American male presented to the clinic in October 2023 with a pruritic eruption on the trunk, upper extremities, and face. He recounted that one week prior to the onset of symptoms, he had consumed a store-bought sandwich containing cooked shiitake mushrooms in South Florida, followed by a salad with raw shiitake mushrooms during a subsequent trip to New York City. The eruption progressively worsened over the next three days, leading to his consultation at our facility. Past medical history included hypothyroidism for which he takes levothyroxine and dilated cardiomyopathy for which he takes sacubitril/valsartan, spironolactone, and empagliflozin. The patient had no prior history of cutaneous disorders. He attributed allergic reactions of unknown type to citrus fruit and emtricitabine/tenofovir. Prior surgical history included rectal surgery of an unknown type. The patient denied any history of alcohol or tobacco use.

**Figure 1:**
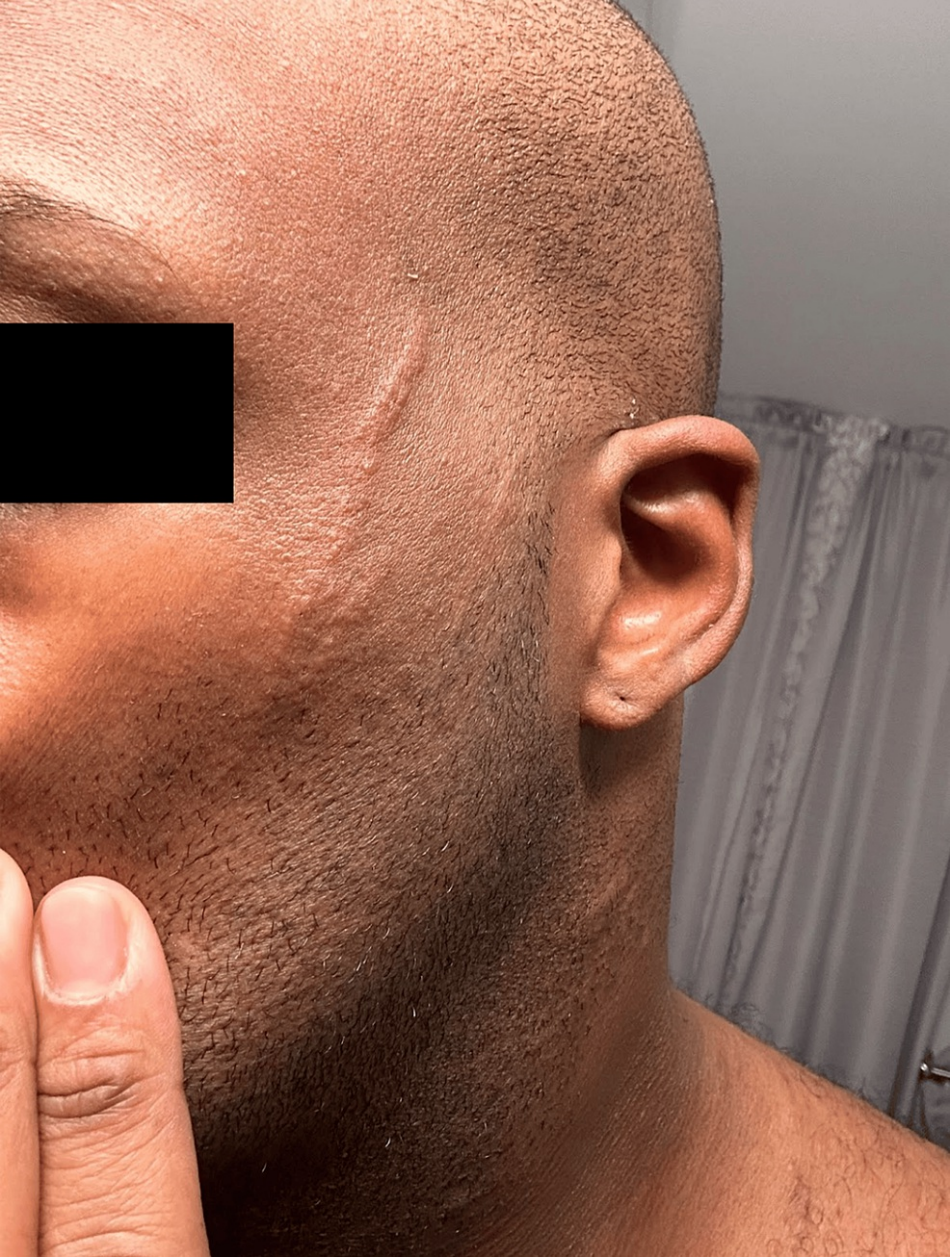
Two parallel, elevated, non-tender, linear lesions prominently visible on the left cheek.

**Figure 2:**
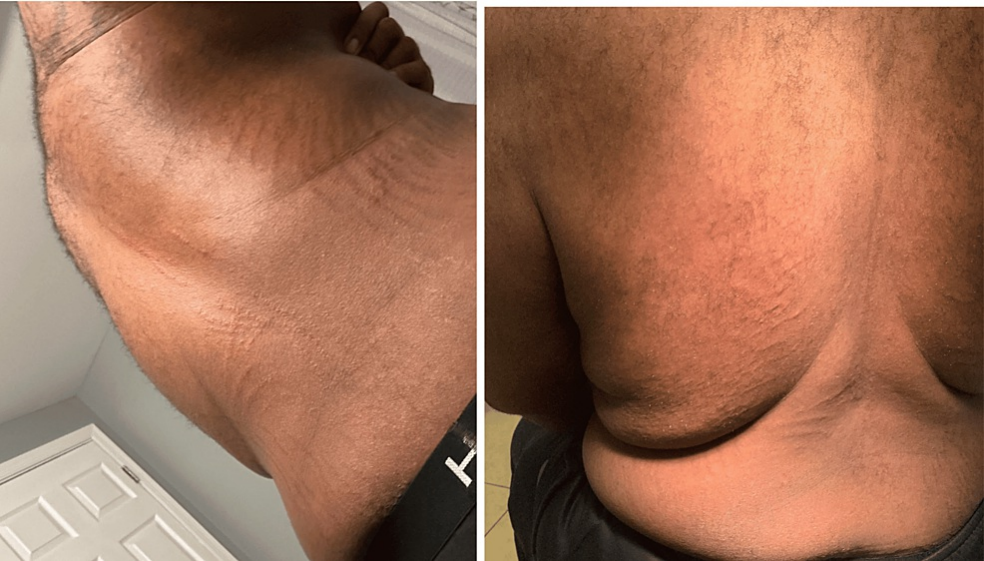
The classic "whip-like" raised, hyperpigmented, grouped curvilinear streaks and plaques of flagellate erythema, distributed across the posterior and right lateral trunk.

**Figure 3:**
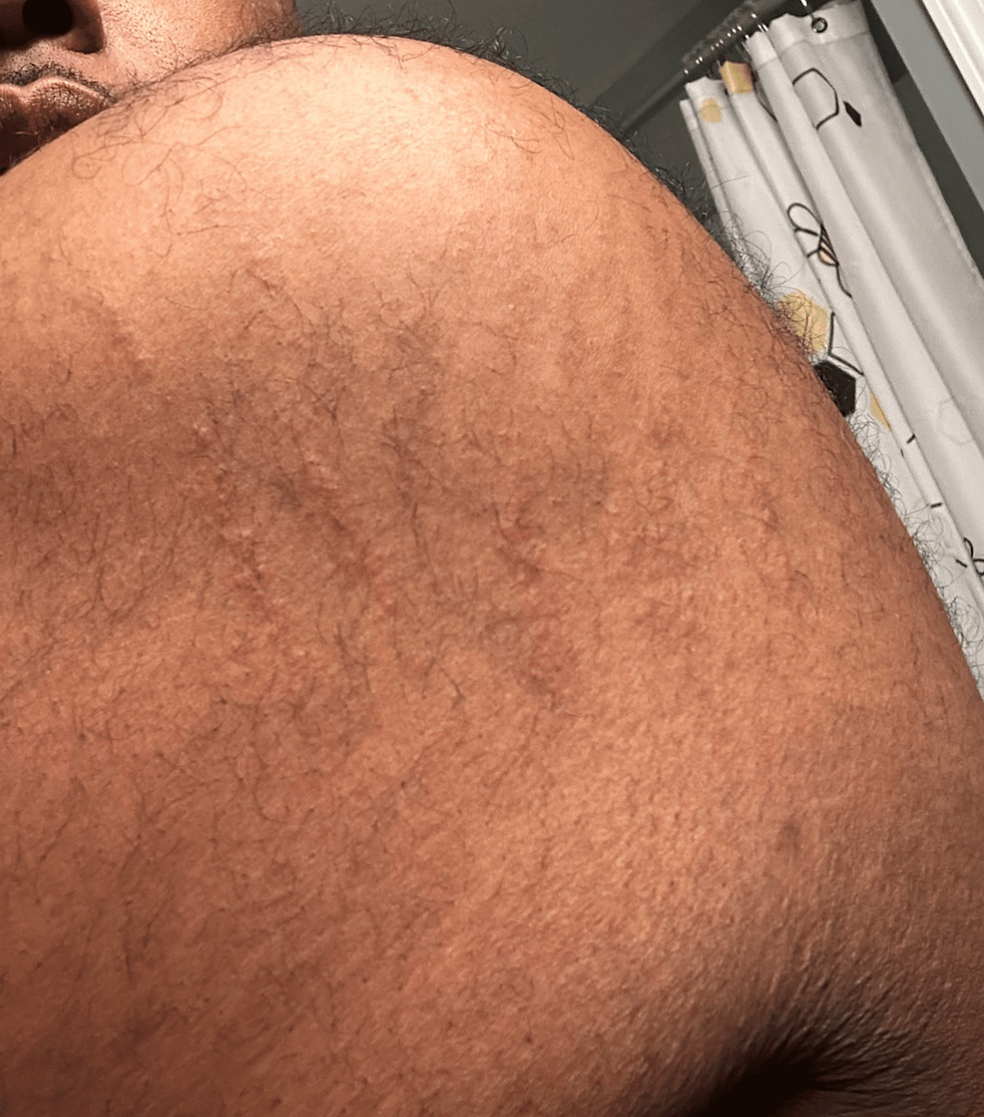
Multiple, parallel, raised, erythematous, linear streaks on the left upper extremity.

Initial physical examination revealed a multifocal cutaneous eruption of multiple non-tender, hyperpigmented, and grouped curvilinear plaques and streaks distributed across the left face (Figure 1), lateral and posterior trunk (Figure 2), and bilateral upper extremities (Figure 3). No mucosal lesions were appreciated. Based on the patient’s clinical presentation, dietary history, and lack of systemic symptoms, a diagnosis of flagellate erythema was made. Given the nonspecific histopathologic findings associated with this dermatitis, flagellate erythema remains a predominantly clinical diagnosis [2,5]. The goals of treatment were targeted at symptomatic relief with a two-week trial of topical corticosteroids and oral antihistamines. Post-treatment evaluation revealed resolution of pruritus and skin changes, and the patient was lost to subsequent follow-up.

## Discussion

Flagellate erythema is a rare cutaneous eruption that should be kept in the differential diagnosis for dermatologists and primary care physicians, especially as mushrooms become a more prominent part of many people’s diets. While the exact pathogenesis remains unclear, cases involving shiitake mushrooms are thought to be related to the thermolabile polysaccharide lentinan, a β-glucan compound derived from the mycelium of shiitake mushrooms. Inadequate cooking of these mushrooms results in a decreased polysaccharide breakdown, and its ingestion is postulated to precipitate a direct toxic reaction and resulting dermal eruption [6]. Although not definitive, it is suggested that lentinans induce the secretion of inflammatory cytokines, including interleukin-1, which in turn leads to vasodilation, hemorrhage, and rash formation [1]. This is due to the release of vasodilatory mediators, such as nitrous oxide and prostaglandins as a part of the body’s innate immune response. There are multiple theories as to how the lesions appear, including being induced by scratching or appearing after scratching via the Koebner phenomenon, but the evidence is conflicting [7].

Flagellate erythema can also be associated with bleomycin chemotherapy, dermatomyositis, and adult-onset Still’s disease. The reaction with bleomycin is commonly a dose-dependent reaction that may be related to the cytotoxic effect of the medication or a variety of inflammatory and pigmentation changes that can be induced as a result of anti-cancer activity [8]. Cases of idiopathic flagellate dermatitis have also been seen in conjunction with the typical dermatoses found in dermatomyositis (e.g., Gottron’s papules, heliotrope rash, and shawl sign), but it is only found to affect about 5% of patients [9]. With flagellate erythema in patients with dermatomyositis, the lesions are typically erythematous and localized to the trunk and proximal extremities, while bleomycin-induced flagellate dermatitis appears more brown and less inflammatory as the markings are thought to be related to melanosome activity in keratinocytes [9]. Flagellate dermatitis can also be seen in adult-onset Still’s disease, but the more typical rash of this disease is a non-pruritic salmon-colored maculopapular rash. Adult-onset Still’s disease is also typically characterized by symptoms, such as fever and arthralgias, which can aid in the differential diagnosis of what is causing the flagellate dermatitis. 

Histopathology is nonspecific, with biopsies chiefly demonstrating acute interface dermatitis and epidermal spongiosis, occasionally present in combination. Additional findings include papillary dermis edema and perivascular lymphocytic and eosinophilic inflammatory infiltrate [10]. Interestingly, the presence of post-inflammatory pigment alteration characterized by the presence of melanophages within the papillary dermis was only recorded in cases attributed to bleomycin administration [10].

## Conclusions

The clinical diagnosis of flagellate erythema hinges on the recognition of its hallmark pruritic and erythematous whiplash-like markings in conjunction with a detailed patient history. Proper identification relies upon the investigation of symptom onset and progression in relation to dietary changes and initiation of any new medications. The role of biopsy as a diagnostic tool is limited due to the nonspecific nature of histological findings. Most cases are self-limiting, with initial eruptions lasting up to three weeks. The aim of treatment should be symptomatic relief via topical corticosteroids and oral antihistamines, which may alleviate associated pruritus and skin changes. In instances linked to undercooked shiitake mushrooms, patient education on avoiding this trigger is crucial. Given the varying degrees of post-inflammatory hyperpigmentation that have been reported, further research is needed to elucidate its impact across the full spectrum of skin types. This lack of data underscores the imperative for further investigation in this domain to enhance dermatological understanding and management of such cases.

## References

[1] Mendonça CN, Silva PM, Avelleira JC, Nishimori FS, Cassia Fde F (2015). Shiitake dermatitis. An Bras Dermatol.

[2] Czarnecka AB, Kreft B, Marsch WCh (2014). Flagellate dermatitis after consumption of Shiitake mushrooms. Postepy Dermatol Alergol.

[3] Boels D, Landreau A, Bruneau C (2014). Shiitake dermatitis recorded by French Poison Control Centers - new case series with clinical observations. Clin Toxicol (Phila).

[4] Pakran J, AlFalasi AA, Al Hammadi AH (2017). Flagellate erythema as manifestation of food hypersensitivity. J Derm Dermatol Surg.

[5] Wu K, de Menezes S, Robinson A (2022). Flagellate erythema: a case of shiitake dermatitis and review of pathogenesis. EMJ Allergy Immun.

[6] Nguyen AH, Gonzaga MI, Lim VM, Adler MJ, Mitkov MV, Cappel MA (2017). Clinical features of shiitake dermatitis: a systematic review. Int J Dermatol.

[7] Cullingham K, Kost G (2021). A case of bleomycin-induced flagellate dermatitis: a case report. SAGE Open Med Case Rep.

[8] Verma P, Rajaram S, Heda A (2022). Bleomycin-induced flagellate dermatitis: revisited. Cureus.

[9] Mai S, Mansouri S, Benzekri L, Senouci K (2019). Severe flagellate erythema in idiopathic dermatomyositis. BMJ Case Rep.

[10] Ching D, Wood BA, Tiwari S, Chan J, Harvey NT (2019). Histological features of flagellate erythema. Am J Dermatopathol.

